# Predictive accuracy of intubation scores and parameters for double lumen tube placement in elective thoracic surgery: a prospective study

**DOI:** 10.1186/s12871-026-04016-2

**Published:** 2026-06-11

**Authors:** Mesher Ensarioğlu, Mehtap Tunç, Ali Alagöz, Hilal Sazak

**Affiliations:** 1 Department of Anesthesiology and Reanimation, Ankara Gülhane Training and Research Hospital, University of Health Sciences, Ankara, Turkey; 2 Department of Anesthesiology and Reanimation, Ankara Atatürk Sanatorium Training and Research Hospital, University of Health Sciences, Ankara, Turkey

**Keywords:** Airway management, Difficult intubation, Double lumen tube intubation, Risk assessment, And Thoracic surgery

## Abstract

**Background:**

Standard predictors (Mallampati score, Wilson Risk Score, airway measurements) validated for single-lumen tubes might not apply to double-lumen tubes (DLT). The aim of this study is to evaluate whether these predictors can identify challenging DLT intubations.

**Material and methods:**

In this prospective study, 200 adult patients undergoing elective thoracic surgery and intubated with DLT were enrolled. Preoperative airway assessments included Mallampati score, Wilson Risk Score, Cormack–Lehane Classification Score (CLS), body mass index (BMI), neck circumference (NC), neck and head mobility, Interincisor distance (IID), Sternomental distance (SMD), thyromental distance (TMD), and the ratio of height to TMD (RHTMD), as well as the NC to TMD ratio were performed. Intubation difficulty was measured using the Intubation Difficulty Scale (IDS). Based on IDS, patients were grouped as easy or difficult intubation, and the effectiveness of the criteria mentioned above in predicting intubation difficulty was assessed.

**Results:**

Intubation difficulties occurred in 36 out of 200 patients (18%). There were no significant demographic differences between the easy and difficult intubation groups. The difficult intubation group showed higher Mallampati, Wilson, and CLS scores. They also had shorter SMD and TMD, smaller IIDs, and a higher height-to-TMD ratio (*p <* 0.01). New DLT-specific cut-off values (SMD 15 cm, TMD 8 cm, IID 3.5 cm) ​​were established. Only the Cormack-Lehane grade independently predicted difficulty. Patients with difficult intubation required additional interventions. Other measures differed between groups but lost significance in regression analysis.

**Conclusion:**

The Cormack–Lehane grade was the only independent predictor of difficulty in intubation with DLT. The new DLT-specific cutoff values suggest that DLT intubation might require its own customized assessment criteria. The proposed new cutoff values could make risk stratification more accurate. Combining scoring and measurements may improve preparation for difficult DLT intubations.

## Introduction

Airway management is a cornerstone of anesthetic practice. Endotracheal intubation is one of the most common procedures used to maintain and protect the airway during elective or emergency interventions [1]. One lung ventilation (OLV) is a technique often performed using a double-lumen tube (DLT) to isolate the lung being operated on and ensure proper ventilation of the other lung. DLTs are designed to be longer and stiffer than single-lumen tubes (SLTs), making intubation more difficult [2].

Difficulty encountered by a conventionally trained anesthesiologist in ventilating the upper airway with a face mask, in tracheal intubation, or both, is defined as a difficult airway by the American Society of Anesthesiologists (ASA) [3]. Methods for managing difficult airways, such as videolaryngoscopy or flexible bronchoscopy, differ based on the expected level of difficulty. More invasive interventions may be necessary in cases of unexpectedly difficult airways, including surgical procedures like tracheostomy. Therefore, estimating the potential for a difficult airway is of utmost importance before intubation.

Parameters influencing difficult airway management have been an area of interest, as predicting potential difficulties enables better preparation for interventions. These parameters include Mallampati score (MS), body mass index (BMI), neck circumference (NC), neck and head mobility, thyromental distance (TMD), height (TMH), Sternomental distance (SMD), hyomental distance (HMD), HMD in normal standing (HMDn), HMD in head extension (HMDe), Ratio of height to TMD (RHTMD), NC to TMD ratio, and Interincisor distance (IID). Scoring systems that combine different sets of these parameters are often used [4–6].

Mallampati score and the Wilson Risk Score (WRS) remain the most commonly used preoperative scoring systems, with WRS calculating a score based on weight, jaw mobility, retrognathia, and incisor evaluation [7]. Scoring systems relying on laryngoscopic views also exist, such as the Cormack-Lehane Classification Score (CLS), which assesses the view obtained during direct laryngoscopy, primarily focusing on the visibility of the glottis and laryngeal structures [8]. Postoperative intubation difficulty assessment tools are also available, including the Intubation Difficulty Scale (IDS), which considers the number of attempts, additional maneuver requirements, use of alternative techniques and the CLS [9]. The mentioned scoring systems and parameters have been routinely used for SLT, and current studies support their validity and reliability. However, studies on difficulty assessment for intubation with DLT remain limited, and no known risk model was available at the time of this study.

Our study hypothesized that, because of the structural differences in the DLT, there may be variations in the parameters and cut-off values used to predict difficult intubation. Our goal was to determine which parameters currently used to predict difficult intubation with SLT might be more suitable for assessing the risk involved in managing difficult airways with DLT.

## Material and methods

The study was conducted after approval from the Ankara Atatürk Sanatorium Training and Research Hospital local Ethics Committee (Approval Date 11/10/2023, decision number 2012-KAEK-15/2822). The study complied with the Declaration of Helsinki and was managed after informed consent was obtained from all participants. It was designed as a prospective observational cohort study, to be carried out at a tertiary care hospital specializing in pulmonary medicine and thoracic surgery. The study commenced on 06/11/2023 with patient admission and concluded on 03/18/2024 after reaching an adequate patient count.

The study’s population consisted of patients being admitted for elective thoracic surgery. Written and informed consent was taken from patients in the study before any surgical procedure, at the time of admission to the thoracic surgery ward for preoperative anesthesia evaluation. Patients aged 18 years or older scheduled for elective thoracic surgery requiring DLT intubation and classified as American Society of Anesthesiologists (ASA) physical status I, II, or III were eligible for inclusion. Patients were excluded if they had a history of thoracic surgery, head and neck tumors, prior head and neck surgery, or radiotherapy to the head and neck region. Patients with inadequate or unavailable medical histories and those who declined to provide informed consent were also excluded. For patients undergoing multiple procedures during the study period, only the initial admission was recorded, and subsequent admissions were excluded from the analysis.

Demographic data of the patients, including height, weight, gender, the type of surgery, any comorbidities, and, if present, previous difficult intubation history, were recorded. Parameters for evaluating difficult intubation, including BMI, SMD, TMD, TMH, HMDn, HMDe, IID, NC, NC/TMD, and height/TMD, were documented. For scoring systems, MS and CLS results were noted.

Jaw subluxation, neck mobility assessment, prominent upper teeth presence, upper lip bite test (ULBT) were recorded and used as additional parameters. Afterwards, an assessment of difficult intubation by WRS was performed, with a score greater than two considered indicative of difficult intubation. For patients with a high anticipated risk of difficult mask ventilation, supraglottic airway difficulty, high aspiration risk, rapid desaturation risk and additional assessment for awake intubation was conducted.

The primary outcome was the identification of preoperative airway assessment parameters independently predictive of difficult double-lumen tube (DLT) intubation. Secondary outcomes included the incidence of difficult DLT intubation, the diagnostic performance of bedside airway measurements, procedural characteristics (number of attempts, total and successful intubation times), and the incidence of intubation-related complications.

MS was assessed with the patient seated, mouth maximally open, and tongue protruded without phonation. Class I indicates full visibility of the soft palate, uvula, and tonsillar pillars; Class II, visibility of the soft palate and uvula; Class III, visibility of the soft palate and the base of the uvula only; and Class IV, visibility of the hard palate alone. Classes III and IV were considered predictive of difficult intubation. CLS was assessed during direct laryngoscopy at the first intubation attempt, in its modified form with five grades: Grade 1, full view of the glottis; Grade 2a, partial view of the glottis; Grade 2b, only the arytenoids or posterior vocal cords visible; Grade 3, only the epiglottis visible; and Grade 4, neither the glottis nor the epiglottis visible. Grades 2b and above were considered indicative of difficult laryngoscopy.

WRS was performed with five parameters; body weight, head and neck mobility, jaw movement (including subluxation), retrognathia, and buck teeth. Each parameter was graded from 0 to 2. Total scores range from 0 to 10, with a score greater than 2 considered predictive of difficult intubation. IDS was calculated after the intubation attempt as the sum of seven components: (N1) number of additional attempts beyond the first; (N2) number of additional operators beyond the first; (N3) number of alternative techniques used (stylet, bougie, videolaryngoscopy, or fiberoptic bronchoscopy); (N4) Cormack–Lehane grade minus 1, using the first attempt as reference; (N5) need for increased lifting force during laryngoscopy; (N6) need for external laryngeal pressure; and (N7) vocal cord adduction at the time of tube passage. An IDS score of 0 indicates easy intubation, 1–5 indicates slight difficulty, and greater than 5 indicates difficult intubation.

All patients were monitored according to ASA standards, with initial monitoring performed in the supine position using electrocardiogram, pulse oximeter, and non-invasive blood pressure measurement. Before anesthesia induction, IV access was established with midazolam 0.03 mg/kg administered, followed by three minutes of preoxygenation. Anesthesia induction was then initiated with 2 mg/kg propofol and 1 mcg//kg fentanyl administered intravenously. For patients who could be ventilated via a mask bag, IV rocuronium 0.6 mg/kg was given before the intubation attempt. Face-mask ventilation was successful in all patients, who consequently all received rocuronium; no patient required deviation for an inability to ventilate. Patients identified preoperatively as being at high risk of difficult mask ventilation were evaluated for awake intubation, and had face-mask ventilation been inadequate after induction, the difficult airway algorithm for one-lung ventilation would have been followed.

After intubation with a Macintosh laryngoscope and DLT, anesthesia maintenance was continued with 2–2.5% sevoflurane, 0.05–2 mcg/kg/min remifentanil, and 0.01–0.012 mg/kg rocuronium. Perioperative blood pressure monitoring and blood gas analyses were performed via arterial cannulation. Direct laryngoscopy with a Macintosh blade was used for the first intubation attempt in all patients. An attempt was defined as the introduction of the laryngoscope blade into the oropharynx, regardless of whether tube passage was attempted. A maximum of three intubation attempts was permitted before escalation to rescue techniques. Between attempts, bag-mask ventilation with 100% oxygen was performed to maintain peripheral oxygen saturation above 95%, and external laryngeal manipulation or head repositioning was allowed at the operator's discretion.

For patients with anticipated difficult intubation, a difficult airway cart was prepared. In cases of unexpectedly difficult intubation, the algorithm for difficult airway management during one-lung ventilation was followed: mask or supraglottic airway ventilation was prioritized to maintain oxygenation, followed by a reattempt at DLT placement with a change in blade size or type and/or the introduction of an adjunct device. If DLT placement remained unsuccessful, a single-lumen tube was placed, and subsequent exchange to a DLT via an airway exchange catheter was attempted; if exchange was not feasible, one-lung ventilation was achieved using a bronchial blocker. All attempts were performed by the same consultant anesthesiologist with dedicated experience in thoracic anesthesia. Another anesthesiologist observed and recorded the total number of intubation attempts, the number of intubation performers, the need for additional techniques and devices, jaw elevation, laryngeal pressure requirement, CLS, the position of the vocal cords, and the total duration of intubation.

For the assessment of difficult intubation, an Intubation Difficult Score (IDS) was calculated after the intubation attempts, and patients with a score above five were classified as having difficult intubation. Hypertension was defined as a systolic blood pressure above 160 mmHg or the requirement for antihypertensive medication. Hypotension was defined as a blood pressure drop of 20% or more compared to the baseline value. A saturation of 90% or lower was considered desaturation.

The size of the DLT was determined according to the patients’ height and sex, with 41Fr used for males above 170 cm and 39Fr for those below 170 cm. A cutoff of 160 cm was used for female patients, with 37Fr and 35Fr selected, respectively. All patients were intubated with a left-sided double-lumen endobronchial tube (Rüsch Bronchopart®, Teleflex Medical, Athlone, Ireland). The confirmation of intubation was performed by fiberoptic bronchoscopy. Additionally, in cases where bougies could not be reliability used, fiberoptic bronchoscopy was also utilized for guidance. Isolated ventilation and clamping the two lumens separately were subsequently used to assess adequate ventilation and lung isolation [10].

For patients with anticipated difficult intubation, an anesthesia cart with additional equipment was prepared, which included blades of different types and sizes, a McCoy laryngoscope blade, a McGrath videolaryngoscope, SLT, bougies, airway exchange catheters, bronchial blockers, and a fiberoptic bronchoscope. For patients with unexpected difficult airway, supraglottic airway devices, a large mask, and a face mask bag ventilator were readily available in the operating room. For reintubation attempts, different blade types and tube sizes were usually used. If additional failure occurred, intubation with guides, including a bougie, was performed. In cases of failure after the aforementioned methods, SLT was preferred to be later exchanged for DLT using airway exchange catheters.

### Statistical analysis

Patients’ data was initially recorded in Microsoft 365 Excel for evaluation of misinputs and missing data if present. Afterwards, the data was transferred to IBM SPSS Statistics for Windows Version 30 for future analysis. Kolmogorov–Smirnov test and histograms were used to estimate the parametric distribution. After confirming the distribution, parameters were presented with mean and standard deviation, or median with 25th and 75th percentiles according to distribution pattern. Nominal parameters were compared with Chi-Square test, while scale parameters were investigated with Pearson correlation. Independent samples T-test was utilized for comparison between parameters among difficult and easy intubation groups. For parameters with statistical significance, area under the curve (AUC) analysis was performed with Youden Index utilization for possible new cut-off values. *P* values of or below 0.05 were accepted as statistically significant.

Regarding patient sample size analysis, the literature review revealed a difficult intubation rate of 9–10% with SLT, while DLT was associated with a difficult intubation rate of 17% [4, 11]. Based on current literature to detect a difference in intubation difficulty with 90% power and a 5% type I error, at least 170 patients were needed. Accounting for a 15% loss due to intraoperative and postoperative factors and potential data loss, a total of 200 patients were chosen as the target sample size.

## Results

**Fig. 1:**
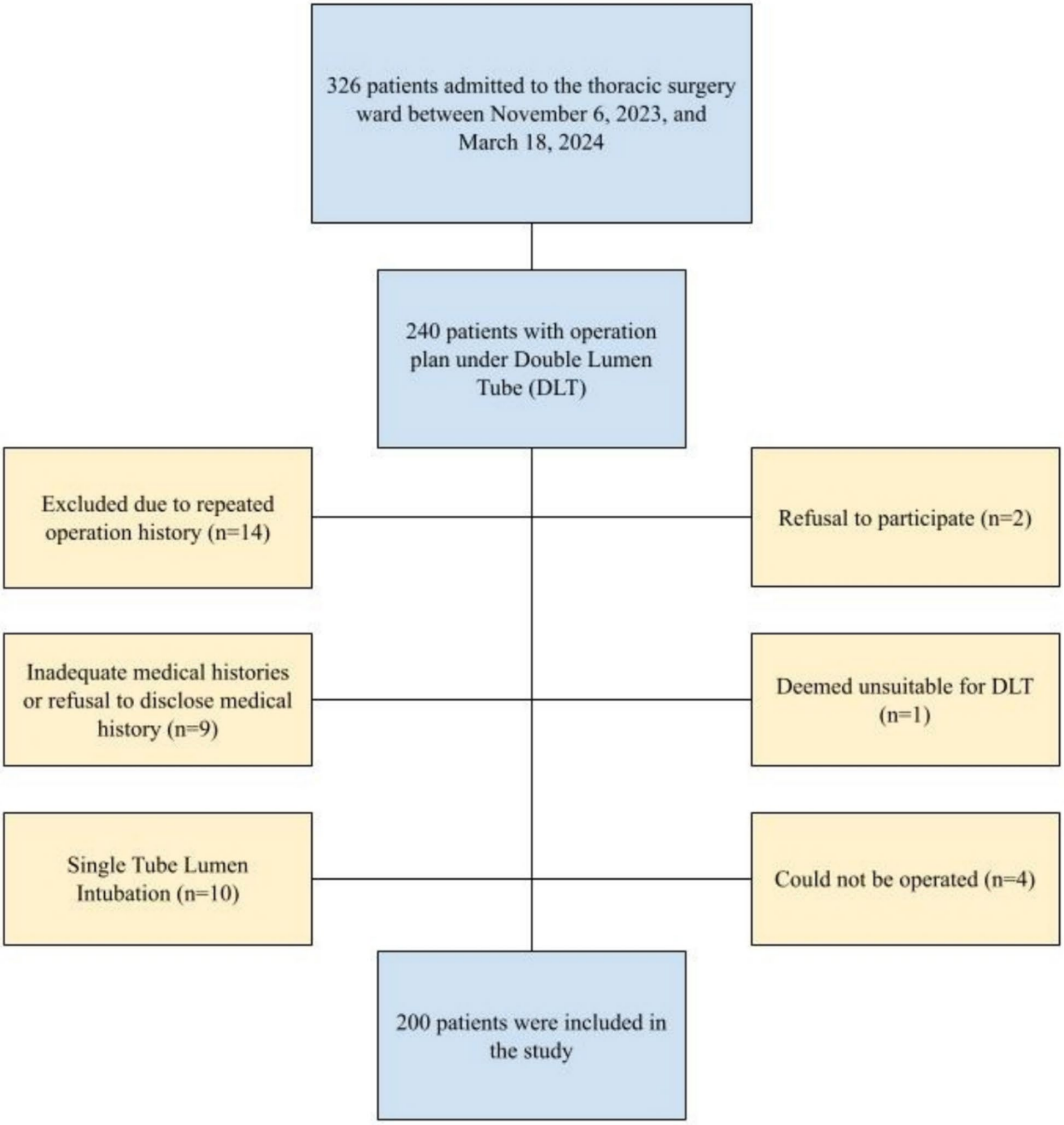
Patient evaluation flow chart

A total of 326 patients admitted to the thoracic surgery ward were evaluated in the study between November 6, 2023, and March 18, 2024. Eighty-six patients were excluded from the study due to non-surgical admission, which included follow-up evaluations and other minimally invasive procedures. Of the remaining 240 patients, fourteen were excluded due to repeated surgical interventions, nine were excluded because of inadequate medical histories, and ten were excluded because of operations planned using SLT. Two patients refused to participate; one patient was later deemed unsuitable for DLT, and four patients could not be operated on due to underlying medical comorbidities after initial evaluation. After excluding these patients, the study population was deemed complete when two hundred patients were reached (Fig. 1).

The mean age of the patients was 55.24 (± 15.53) years, and the majority of the patients were male (*n* = 142, 71%). The mean height was 169.5 (± 8.68) cm, and weight was 76.52 (± 12.97) kg, with an average BMI of 26.68 (± 4.68) kg/m^2^. The most commonly observed comorbidities were hypertension (*n* = 58, 29%), diabetes mellitus (*n* = 35, 17.5%), and coronary artery disease (*n* = 34, 17%). Seventy-five patients (37.5%) were either active or ex-smokers, with the remaining 125 (62.5%) being nonsmokers. Thoracotomy was performed in 69 (34.8%) patients, while video-assisted thoracoscopic surgery (VATS) was utilized for 129 (65.2%) patients. Regarding comorbidities, demographic characteristics, and surgical methods, no statistically significant difference was observed between patients with normal intubation (*n* = 164) and those with difficult intubation (*n* = 36) (Table 1).

**Table 1:** Demographic characteristics, comorbidities and surgical approach

**Parameters** (n, %)		**Intubation Difficulty According to IDS**			***P* Value**
		Normal (*n* = 164)	Difficult (*n* = 36)	Total (*n* = 200)	
Age (years, ± SD)		55.31 (15.91)	54.89 (13.84)	55.24 (15.53)	0.883
Height (cm, ± SD)		169.81 (8.75)	168.89 (8.42)	169.65 (8.68)	0.565
Weight (kg, ± SD)		76.02 (13.05)	78.75 (12.51)	76.52 (12.97)	0.255
Body Mass Index (kg/m^2^, ± SD)		26.48 (4.8)	27.6 (4.02)	26.68 (4.68)	0.195
Sex	*Male*	117 (71.3)	25 (69.4)	142 (71)	0.82
	*Female*	47 (28.7)	11 (30.6)	58 (29)	
Diabetes Mellitus	*No*	135 (82.3)	30 (83.3)	165 (82.5)	0.884
	*Present*	29 (17.7)	6 (16.7)	35 (17.5)	
Hypertension	*No*	111 (67.7)	31 (86.1)	142 (71)	0.027^*^
	*Present*	53 (32.3)	5 (13.9)	58 (29)	
Chronic Obstructive Lung Disease	*No*	135 (82.3)	32 (88.9)	167 (83.5)	0.459^*^
	*Present*	29 (17.7)	4 (11.1)	33 (16.5)	
Asthma	*No*	154 (93.9)	34 (94.4)	188 (94)	N/A
	*Present*	10 (6.1)	2 (5.6)	12 (6)	
Obstructive Sleep Apnea	*No*	163 (99.4)	36 (100)	199 (99.5)	N/A
	*Present*	1 (0.6)	0 (0)	1 (0.5)	
Chronic Obstructive Lung Disease	*No*	135 (82.3)	31 (86.1)	166 (83)	0.807^*^
	*Present*	29 (17.7)	5 (13.9)	34 (17)	
Chronic Renal Disease	*No*	162 (98.8)	35 (97.2)	197 (98.5)	0.450^*^
	*Present*	2 (1.2)	1 (2.8)	3 (1.5)	
Smoking History	*No*	105 (64)	20 (55.6)	125 (62.5)	0.342
	*Present*	59 (36)	16 (44.4)	75 (37.5)	
Extrathoracic Malignancy	*No*	152 (92.7)	31 (86.1)	183 (91.5)	0.2
	*Present*	12 (7.3)	5 (13.9)	17 (8.5)	
Surgical Method	*Thoracotomy*	60 (36.8)	9 (25.7)	69 (34.8)	0.211
	*VATS*	103 (63.2)	26 (74.3)	129 (65.2)	

*SD* Standard Deviation, *VATS* Video-Assisted Thoracic Surgery. Smoking History definition included both ex and active smokers. *IDS* Intubation Difficulty Score, *BMI* Body Mass Index

^*^Comparisons between groups were made by Fisher's Exact Test

Regarding difficult intubation assessment scoring, patients had an almost even distribution of ASA grade, with 102 patients classified as ASA II and 98 patients as ASA III, with no significant difference between groups regarding difficult intubation (*p* = 0.216). Patients in the difficult intubation group had higher Mallampati scores and CLS compared to those in the normal intubation group (*p* values of 0.016 and 0.001, respectively). The upper lip bite test did not differ between groups (*p* = 0.643). WRS, as an overall score, was higher among patients with difficult intubation, with a median of 2 (1–4) compared to a median of 1 (1–2) in the normal intubation group (*p* = 0.001). In the subgroup analysis of WRS, retrognathia and buck teeth were more prominent in the difficult intubation group (*p* = 0.001), while no statistically significant differences were observed in other WRS components. Regarding measurements, SMD, TMD and IID were higher in the normal intubation group (17.63 ± 2.09 cm to 16.22 ± 2.45 cm, 8.6 ± 1.18 cm to 7.76 ± 1.53 cm, and 4.45 ± 0.71 cm to 4.03 ± 0.76 cm, respectively, *p* values of 0.001), while RHTMD was higher in the difficult intubation group (20.08 ± 2.64 to 22.52 ± 43, *p* value of 0.002). Other measurements, including TMH, HMDe, HMDn, NC, Hyomental Distance Ratio, and the NC to TMD ratio, did not vary statistically between groups (Table 2).

**Table 2:** Scoring systems and measurements for intubation difficulty assessment

Intubation Difficulty Assessment Parameters (n, %)		Intubation Difficulty According to IDS			*P* Value
		Normal (*n* = 164)	Difficult (*n* = 36)	Total (*n* = 200)	
American Society of Anesthesia Score	*2*	87 (53)	15 (41.7)	102 (51)	0.216
	*3*	77 (47)	21 (58.3)	98 (49)	
Mallampati Score	*1*	45 (27.4)	4 (11.1)	49 (24.5)	0.016
	*2*	78 (47.6)	14 (38.9)	92 (46)	
	*3*	27 (16.5)	13 (36.1)	40 (20)	
	*4*	14 (8.5)	5 (13.9)	19 (9.5)	
Cormack-Lehane Classification Grade	*1*	93 (56.7)	3 (8.3)	96 (48)	0.001
	*2a*	57 (34.8)	10 (27.8)	67 (33.5)	
	*2b*	13 (7.9)	13 (36.1)	26 (13)	
	*3*	1 (0.6)	10 (27.8)	11 (5.5)	
Upper Lip Bite Test Class	*1*	79 (48.2)	16 (44.4)	95 (47.5)	0.643
	*2*	65 (39.6)	17 (47.2)	82 (41)	
	*3*	20 (12.2)	3 (8.3)	23 (11.5)	
Wilson Risk Score and Parameters (Median, 25-75th)	*Weight*	0 (0–0)	0 (0–0)	0 (0–0)	0.466
	*Mobility*	0 (0–0)	0 (0–1)	0 (0–1)	0.119
	*Jaw*	1 (0–1)	1 (1–1)	1 (0–1)	0.251
	*Subluxation*	0 (0–1)	0 (0–1)	0 (0–1)	0.066
	*Retrognathia*	0 (0–0)	0 (0–1)	0 (0–1)	0.001
	*Buck Teeth*	0 (0–0)	0 (0–1)	0 (0–0)	0.001
	*Total*	1 (1–2)	2 (1–4)	1 (1–3)	0.001
Measurements (cm, ± SD)					
Sternomental Distance		17.63 (2.09)	16.22 (2.45)	17.38 (2.22)	0.001
Thyromental Distance		8.6 (1.18)	7.76 (1.53)	8.45 (1.29)	0.001
Thyromental Height		5.24 (1.2)	5.06 (1.13)	5.21 (1.19)	0.399
Hyomental Distance (Extension)		5.63 (0.88)	5.22 (1.34)	5.56 (0.99)	0.085
Hyomental Distance (Neutral)		4.33 (0.69)	4.03 (1.09)	4.28 (0.78)	0.113
Neck Circumference		38.8 (3.33)	39.24 (4.03)	38.88 (3.46)	0.491
Interincisor Distance		4.45 (0.71)	4.03 (0.76)	4.38 (0.74)	0.001
The ratio of Height to TMD		20.08 (2.64)	22.52 (4.3)	20.52 (3.14)	0.002
Hyomental Distance Ratio		1.31 (0.13)	1.31 (0.15)	1.31 (0.14)	0.934
The ratio NC to TMD		4.4 (0.37)	4.34 (0.37)	4.39 (0.37)	0.343

*SD* Standard Deviation, *NC* Neck Circumference, *TMD* Thyromental Distance Phi and Cramer V was utilized for comparison between groups. Cormack Lehane, Mallampati, Buck teeth and retrognathia had an effect size of 0.458, 0.227, 0.265 and 0.331, respectively. *IDS* Intubation Difficulty Score

Patients in the difficult intubation group had a higher number of intubation attempts (median of 2 compared to 1), a longer total intubation duration (median of 28 (20–38) seconds compared to a median of 99 (66.5–167.5 s), and a longer time to successful intubation (median of 28 (20–35) seconds compared to 57.5 (35–70) seconds) (*p* = 0.001). Blade change during intubation was more common in the difficult intubation group (*n* = 12, 33.3%) compared to the normal intubation group (*n* = 3, 1.8%). Similarly, the requirement for fiberoptic bronchoscopy was more frequent in the difficult intubation group (*n* = 6, 16.7%) than in the normal intubation group (*n* = 10, 6.1%) (*p* value of 0.034). Other additional interventions did not differ significantly between groups (Table 3).

**Table 3:** Intubation attempts, additional device requirements and complications

**Parameters** (n, %)		**Intubation Difficulty According to IDS**			***P* Value**
		Normal (*n* = 164)	Difficult (*n* = 36)	Total (*n* = 200)	
Attempt Count		1 (1–1)	2 (2–3)	1 (1–1)	0.001
Total Intubation Period (sec)		28 (20–38)	99 (66.5–167.5)	30 (20–50)	0.001
Time to Successful Intubation (sec)		28 (20–35)	57.5 (35–70)	30 (20–40)	0.001
Blade Change	No	161 (98.2)	24 (66.7)	185 (92.5)	0.001
	Required	3 (1.8)	12 (33.3)	15 (7.5)	
Tube Change	No	158 (96.3)	32 (88.9)	190 (95)	0.063
	Required	6 (3.7)	4 (11.1)	10 (5)	
Videolaryngoscopy	No	164 (100)	32 (88.9)	196 (98)	N/A
	Required	0 (0)	4 (11.1)	4 (2)	
Tube Exchanger	No	164 (100)	29 (80.6)	193 (96.5)	N/A
	Required	0 (0)	7 (19.4)	7 (3.5)	
Fiberoptic Bronchoscopy	No	154 (93.9)	30 (83.3)	184 (92)	0.034
	Required	10 (6.1)	6 (16.7)	16 (8)	
Complication Evaluation					
Desaturation	No	162 (98.8)	31 (86.1)	193 (96.5)	0.001^*^
	Present	2 (1.2)	5 (13.9)	7 (3.5)	
Hypertension	No	126 (76.8)	15 (41.7)	141 (70.5)	0.001
	Present	38 (23.2)	21 (58.3)	59 (29.5)	
Lip Injury	No	158 (96.3)	29 (80.6)	187 (93.5)	0.001
	Present	6 (3.7)	7 (19.4)	13 (6.5)	
Oral Tissue Injury	No	149 (90.9)	17 (47.2)	166 (83)	0.001
	Present	15 (9.1)	19 (52.8)	34 (17)	
Sore Throat	No	115 (70.1)	15 (41.7)	130 (65)	0.001
	Present	49 (29.9)	21 (58.3)	70 (35)	
Hoarseness	No	134 (81.7)	18 (50)	152 (76)	0.001
	Present	30 (18.3)	18 (50)	48 (24)	
Difficult Tube Insertion	No	141 (86)	20 (55.6)	161 (80.5)	0.001
	Present	23 (14)	16 (44.4)	39 (19.5)	

Numerical variables were compared with Mann Whitney U test, and presented with median, 25th and 75th percentiles

^*^Comparison between groups were made with Fisher Exact T test. *IDS* Intubation Difficulty Score

All complications and injuries were more common in the difficult intubation group (*p* values of 0.001). These included desaturation (13.9% to 1.2%), hypertension (58.3% to 23.2%), lip injury (19.4% to 3.7%), oral tissue injury (52.8% to 9.1%), sore throat (58.3% to 29.9%), and hoarseness (50% to 18.3%). According to the assessment of the anesthesiology specialist, difficult tube insertion was also more frequently observed in the difficult intubation group (44.4% to 14%, *p* value of 0.001) (Table 3).

**Fig. 2:**
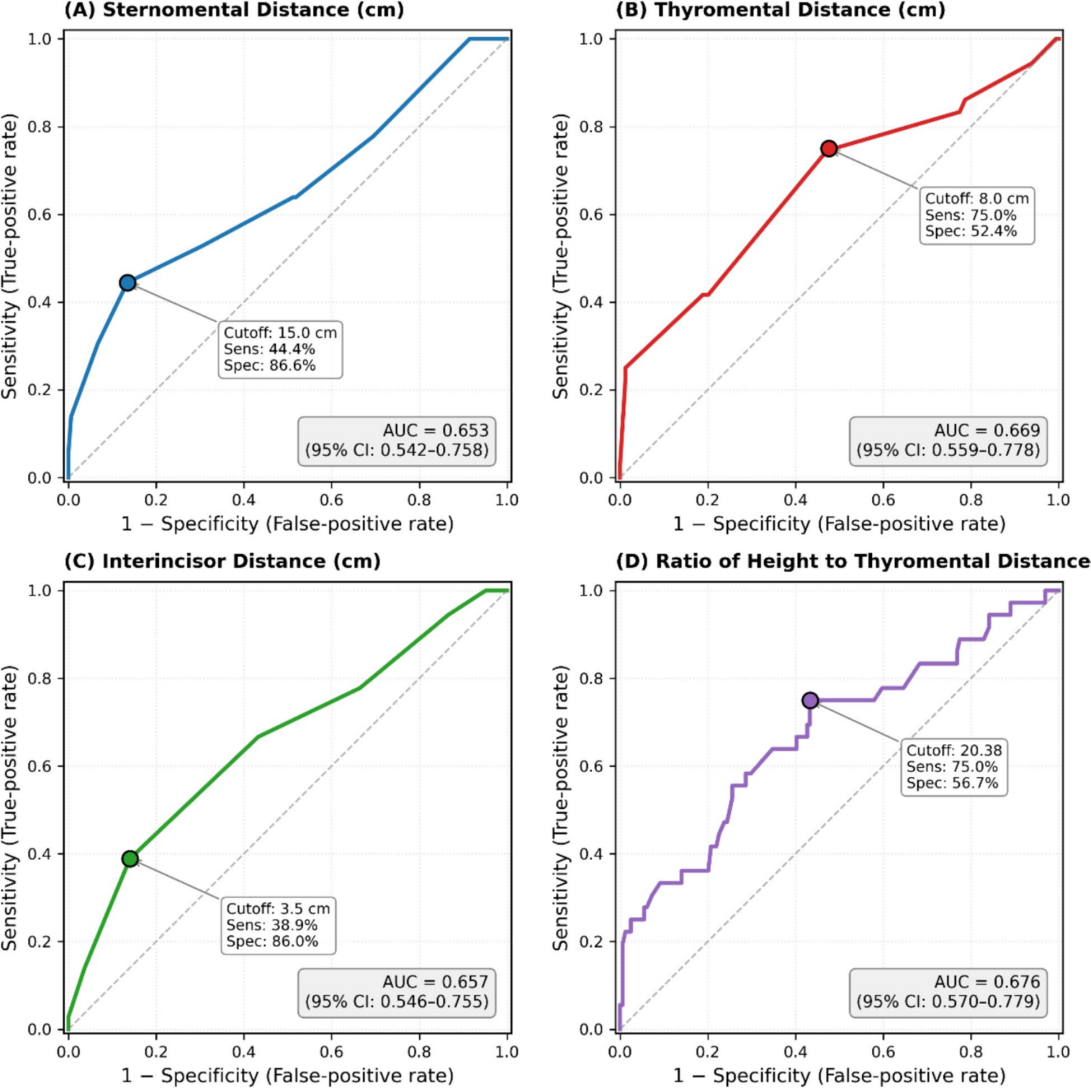
Cut-off values for parameters regarding difficult intubation

Statistical significance measurements were evaluated using the area under the curve (AUC) and then investigated for a cut-off with the Youden Index. In the performed statistical evaluation, SMD, TMD, IID, and RHTMD had statistically significant AUC values (0.653, 0.669, 0.657, and 0.676; with *p* values of 0.006, 0.002, 0.003 and 0.001 respectively). Cut-off values of 15 cm for SMD, 8 cm for TMD, 3.5 cm for IID and 20.38 for RHTMD were observed. The Delong analysis, performed pairwise with the statistically relevant parameters, did not indicate a significant AUC difference between groups (Fig. 2).

A binomial regression analysis model was created to investigate possible independent parameters affecting difficult intubation. Mallampati, CLS, WRS, SMD, TMD, IID and RHTMD were included in the analysis. The model was statistically significant (Chi-square of 71.916 and *p* value of 0.001), had an acceptable Nagelkerke R^2^ (0.495) and classified 88% of cases correctly. It was not considered an overfitted model, with a Hosmer–Lemeshow test value of 0.596. In the model, CLS was observed to be an independent risk factor for difficult intubation with DLT (p:0.001, odds ratio of 6.3). Other parameters, while significant in the former analysis, were not independent risk factors (Table 4). A secondary model was prepared without CLS, to better reflect available bedside evaluation methods. This model had lower Nagelkerke R^2^ (0.211), classified 85% of cases and had a Hosmer–Lemeshow test value of 0.656. In this analysis, RHTMD was observed to be the sole independent predictor factor for difficult airway (p:0.035, odds ratio 1.52) (Table 5).

**Table 4:** Regression analysis of parameters affecting difficult intubation

Parameters	B	Standard Error	Wald	*P* Value	Odds Ratio	95% C.I.for Odds Ratio	
						**Lower**	**Upper**
Mallampati	−0.354	0.312	1.289	0.256	0.702	0.381	1.293
CLS	1.842	0.344	28.641	0.001	6.310	3.214	12.390
WRS	0.084	0.238	0.124	0.725	1.087	0.682	1.733
SMD	−0.307	0.188	2.678	0.102	0.736	0.509	1.063
TMD	0.688	0.633	1.180	0.277	1.989	0.575	6.878
IID	−0.173	0.426	0.165	0.685	0.841	0.365	1.939
RHTMD	0.300	0.227	1.745	0.187	1.350	0.865	2.106
Constant	−10.800	8.777	1.514	0.219			

*CLS* Cormack-Lehane Score, *WRS* Wilson Risk Score, *SMD* Sternomental Distance, *TMD* Thyromental Distance, *IID* Interincisor Distance, *RHTMD* Ratio of height to thyromental distance, *C.I* Confidence Interval

**Table 5:** Regression analysis of parameters affecting difficult intubation with cormack lehane classification excluded

Parameters	B	Standard Error	Wald	*P* Value	Odds Ratio	95% C.I.for Odds Ratio	
						**Lower**	**Upper**
Mallampati	0.268	0.228	1.379	0.240	1.307	0.836	2.043
WRS	0.171	0.186	0.842	0.359	1.186	0.824	1.709
SMD	−0.198	0.147	1.815	0.178	0.821	0.616	1.094
TMD	0.869	0.549	2.508	0.113	2.385	0.813	6.994
IID	−0.372	0.336	1.226	0.268	0.689	0.357	1.332
RHTMD	0.420	0.199	4.468	0.035	1.521	1.031	2.245
Constant	−13.519	7.581	3.180	0.075	0.000		

*WRS* Wilson Risk Score, *SMD* Sternomental Distance, *TMD* Thyromental Distance, *IID* Interincisor Distance, Ratio of height to thyromental distance, *C.I* Confidence Interval

## Discussion

Our study investigated the suitability of the difficult intubation prediction criteria used for single-lumen tubes to determine intubation difficulty for DLT. Significant differences were observed in measurements such as SMD, TMD, IID, and RHTMD in the group undergoing difficult intubation with DLT, and different threshold values ​​were revealed compared to those for difficult SLT intubation. MS, CLS, and WRS were higher in patients with difficult intubation. Notably, prominent upper teeth and retrognathia were two parameters that contributed more to the higher WRS score in the difficult intubation group. CLS grade was identified as an independent factor after accounting for other parameters, and, after excluding CLS, RHTMD was observed to be an independent predictor. However, there was no difference in the difficult intubation group regarding BMI and ULBT.

The standard cutoff values for SMD (< 12.5), TMD (< 6 cm), and IID (< 4 cm) did not align with the results, as our study found new values of 15 cm for SMD, 8 cm for TMD, 3.5 cm for IID, and 20.38 for RHTMD [5, 12]. Excluding RHTMD, these parameters were not independent risk factors for difficult intubation, they were also not superior to each other, justifying their potential use in evaluating difficult intubation for DLT. This also supports the need for establishing new cutoff values for better estimation. Combined with the observation of other measurements being not statistically relevant, these results strengthen the assumption that separate risk assessment modalities are required for DLT compared to SLT in terms of difficult airway.

Longer intubation times, a higher median number of intubation attempts, and the need for blade changes and fiberoptic bronchoscopy during difficult intubation with DLT were observed. Similarly, complications were also more common in the difficult intubation group. Although not an objective assessment, difficult tube insertion, according to the performing anesthesiologist, was another parameter more frequently seen in the difficult intubation group.

Wilson Risk Score, MS, and CLS were all reported to correlate with IDS regarding the estimation of difficult intubation, which aligns with existing literature [7, 13]. In subgroup analysis of WRS parameters, retrognathia and prominent upper teeth were each found to be statistically significant. While IID was associated with IDS, a new cutoff of 3.5 cm was found to be reliable for estimating the likelihood of difficult intubation.

Many studies have been conducted to estimate possible ratios among demographic parameters for difficult airway and intubation assessment. Rao et al., in their prospective study, stated that TMH had higher sensitivity and specificity than the RHTMD ratio in predicting difficult intubation with SLT. An additional observation was that NC to TMD was more reliable than RHTMD [6]. Our study was similar in some aspects of DLT investigation: the RHTMD ratio was statistically significant and remained the sole independent predictor, whereas the NC-to-TMD ratio was not.

Thyromental height was investigated by Palcyznynski et al. in its role regarding DLT intubation difficulty [14]. The study found that only CLS and TMH adequately predicted difficult intubation, whereas MS, TMD, and SMD did not play a statistically significant role. Our study partially agrees with these results: while many parameters, including TMD, SMD, CLS, and IID, differed between groups, only CLS was observed to be an independent factor for difficult intubation.

ULBT is a commonly used evaluation method to assess difficult intubation; however, its role in DLT is debatable. Tang et al., in their study, stated that while it is reliable on its own, when compared with IID, ULBT has lower sensitivity and specificity [15]. Wang et al. also reported similar findings, showing ULBT has low predictive power for difficult intubation [16]. An opposing view was supported by Kar et al.: their study found that ULBT was correlated with MS and thus suggested it as an easy-to-use alternative. However, it was also clarified that, under ideal conditions, both tests should be utilized [8]. Our study found IID to be statistically significant, whereas ULBT did not differ between intubation groups; however, this may partly be attributable to the relatively small number of ULBT Grade 3 patients. Alemayahu et al. reported that ULBT did not predict difficult intubation, but rather, predicted difficult laryngoscopy, and found TMD and RHTMD to be correlated with difficult intubation [17]. Our study presents similar findings, with CLS being the primary risk factor and RHTMD being the sole bedside evaluation that was an independent predictor. 

Obesity not only complicates airway management but also indirectly affects measurements, especially NC and related ratios. Several studies in the literature evaluate the role of obesity, often combining demographic parameters to assess the validity of other measurements. According to two recent studies, the NC to TMD ratio could be preferred over each individual parameter [18, 19]. Pradeep et al. supported the estimation of TMD, NC, and their ratio over SMD for both obese and non-obese populations [20]. There are studies comparing SLT to DLT in terms of obesity, such as the study by Mehta et al., which stated that BMI did not play a significant role in difficult intubation [4]. Çelik et al. reported that, unlike normal assessment criteria, separate cut-offs should be used for obesity, as they reported a cutoff of 50 cm for NC and 6.5 cm for TMD [21]. Our study did not find a correlation with obesity; however, we believe this may be attributable to the overall lower BMI in our sample compared with the literature, with no patients having a BMI over 40 kg/m^2^ in our analysis.

Although the newly identified DLT-specific cut-offs capture a larger proportion of difficult intubations than the SLT-based thresholds, this improvement in sensitivity comes at a substantial cost to specificity, warranting explicit discussion. Using the conventional TMD cut-off of ≤ 6 cm, only 10 of 200 patients in the cohort would have been considered preoperatively at risk of difficult intubation; 8 would have been true positives and 2 false positives, but 28 of 36 truly difficult intubations (77.8%) would have been missed. Raising the threshold to ≤ 8 cm increased the number of patients identified as high-risk to 105, of whom 27 (75%) were difficult, but 78 (74.3%) were false positives. A similar pattern was observed for SMD: the conventional threshold of ≤ 12.5 cm noted only 2 patients and missed 34 of 36 difficult intubations, whereas the proposed ≤ 15 cm threshold flagged 38 patients (16 true positives, 22 false positives). In contrast, the proposed IID cut-off (≤ 3.5 cm) is more stringent than the conventional ≤ 4 cm threshold, prioritizing specificity (86.0% vs 56.7%) at the expense of sensitivity (38.9% vs 66.7%).

These trade-offs have clinical implications. A preoperative risk assessment that may label nearly half of patients as potentially difficult, as seen with new TMD and SMD thresholds, would reduce its discriminative value. Conversely, the higher negative predictive values associated with the new cut-offs (TMD NPV 90.5%, RHTMD NPV 91.2%) suggest that their primary clinical utility may lie in ruling out rather than ruling in difficult intubation, as a patient with TMD > 8 cm or RHTMD < 20.38 has greater than 90% probability of uncomplicated DLT placement. Accordingly, we would caution against using any of these thresholds as a standalone screening tool; rather, they are best interpreted as one component of a multivariable clinical assessment that incorporates Cormack–Lehane grade, Mallampati score, Wilson Risk Score, and clinical history of prior difficult intubation. The observation of multiple parameters affecting difficult intubation with DLT, even though CLS is the only independent risk factor, is supported by the literature. Specialized scoring systems, such as ARNE for evaluations of the head, neck, and throat, exist and incorporate various parameters [22]. As demonstrated in Başpınar et al.’s study, due to the inherent difficulty of head, neck, and throat surgeries, estimating difficult intubation based on a single parameter was not feasible, leading to the use of a combination of WRS, MS, ULBT, and IID [23]. Considering the unique nature of DLT, we can state that a combination of the statistically significant parameters, including measurements of SMD, TMD, RHTMD, and bedside evaluation with WRS and MS, could be utilized in future studies.

This study has some limitations. First, its single-center and tertiary thoracic surgery center nature limits its generalizability. A multi-center study would be beneficial to validate cut-off values and present a robust prediction model. Another limitation was due to possible overfitting of regression models, due to the limited number of difficult intubations, which further supports the need for a multicenter study with more patients. Additionally, selection bias can be considered a significant limitation. However, selection bias was unavoidable because DLT was used for OLV in elective thoracic cases, which naturally exclude emergencies requiring lung isolation (e.g., massive hemoptysis). Nevertheless, due to demographic considerations and calculated parameters, an urgent evaluation for other uses of DLT could not be included in the study.

## Conclusion

It was shown that double-lumen tube intubation for one-lung ventilation has a different risk profile than single-lumen intubation: Cormack–Lehane grade was the only independent factor for difficult DLT intubation, and when it was excluded, RHTMD was the only bedside assessment found to be an independent predictor. Mallampati score, Wilson Risk Score, and bedside measurements (Sternomental, thyromental and interincisor distance) were linked to difficulty but not independently predictive. New cut-offs (SMD 15 cm, TMD 8 cm, IID 3.5 cm, RHTMD 20.38) were established that may better reflect DLT-specific anatomy and could enhance preoperative identification of higher-risk patients. A new scoring system might provide more insight and help ensure proper preparation for DLT.

## Data Availability

The data is available upon reasonable request from the authors, in both raw and edited versions, after approval from the local ethics committee for data sharing.

## Abbreviations

ASA: American Society of Anesthesiologists
AUC: Area Under the Curve
BMI: Body Mass Index
CI: Confidence Interval
CLS: Cormack–Lehane Classification Score
DLT: Double-Lumen Tube
HMD: Hyomental Distance
HMDe: Hyomental Distance in head extension
HMDn: Hyomental Distance in neutral position
IDS: Intubation Difficulty Scale
IID: Interincisor Distance
IV: Intravenous
MS: Mallampati Score
NC: Neck Circumference
NPV: Negative Predictive Value
PPV: Positive Predictive Value
RHTMD: Ratio of Height to Thyromental Distance
SD: Standard Deviation
SLT: Single-Lumen Tube
OLV: One-Lung Ventilation
SMD: Sternomental Distance
TMD: Thyromental Distance
TMH: Thyromental Height
ULBT: Upper Lip Bite Test
VATS: Video-Assisted Thoracoscopic Surgery
WRS: Wilson Risk Score
